# The prospect of genetically engineering natural killer cells for cancer immunotherapy

**DOI:** 10.1242/bio.059396

**Published:** 2022-11-29

**Authors:** Angie Yu Ching Poon, Ryohichi Sugimura

**Affiliations:** School of Biomedical Science, University of Hong Kong, 21 Sassoon Rd, Hong Kong 999077

**Keywords:** CAR-NK, Cancer, Cytokines, Genetic engineering, Immunotherapy, NK cell

## Abstract

The use of natural killer (NK) cells in cancer immunotherapy demonstrates promising potential, yet its efficacy is often limited due to the loss of tumor-killing capacity and lack of specificity *in vivo*. Here, we review current approaches to confer enhanced tumor-killing capacity and specificity by genetic engineering. Increasing sensitivity to cytokines and protecting NK cells from the immune checkpoint endowed sustainability of NK cells in the tumor microenvironment. Transducing chimeric antigen receptor (CAR) in NK cells successfully targeted both hematologic and solid tumors in preclinical models. The use of human pluripotent stem cells as an expandable and genetically amenable platform offers a stable source of engineered NK cells for cancer immunotherapy. We highlight that CAR-NK cells from human pluripotent stem cells are a promising approach for cancer immunotherapy.

## Teaser

**Summary:** We review recent progress in natural killer (NK) cells for cancer immunotherapy, from the basics of NK cell biology to chimeric antigen receptor-transduced NK cell applications.

## Introduction

Natural killer (NK) cells are potent immune effectors in the innate arm that contribute significantly to eliminating tumors and viral-infected cells. They are classically defined as CD3-CD56+ cells and express an array of germline-encoded activating and inhibitory receptors. Activation and inhibitory signals of NK cells are tightly balanced to avoid unwanted destruction.

Activation receptors of NK cells can recognize stress-induced ligands. For example, human NKG2D on NK cells recognizes MHC I chain-related molecules A and B (MICA and MICB), which are aberrantly expressed in various types of tumors (Dhar and Wu, 2018). Meanwhile, inhibitory receptors on NK cells recognize ‘self-ligands’. MHC class I molecules (HLA-C/B/A/E) are expressed ubiquitously on healthy cells but are lost under stress or transformation. Many inhibitory receptors of NK cells [i.e. CD94/NKG2A or killer-immunoglobulin receptors (KIRs)] engage with the MHC class I molecules (Vivier et al., 2008), sending out negative signals from sensing of a ‘self-ligand’. NK cells are only activated when the equilibrium between activating and inhibitory receptors is disrupted, ensuring tolerance to a healthy ‘self’ while effectively targeting transformed cells. The current paradigm believes that the summative strength of signaling determines NK cell reactivity. Simply loss of MHC-I molecules does not always elicit activation (Bern et al., 2019) unless a sufficiently potent activating receptor is engaged (Cerwenka et al., 2001).

Upon activation, NK cells will exert their cytotoxic machinery, which predominately includes (1) expressing death receptor ligands to induce death receptor-mediated apoptosis; and (2) degranulation to secrete perforin and granzymes. Fas ligands (FasLs), a type of death receptor ligand, are upregulated in cytokine-primed human NK cells and induce apoptosis of Fas-expressing tumor cells independent of degranulation-mediated killing (Mori et al., 1997). The binding between Fas-FasL initiates the ‘extrinsic pathway’ of apoptosis. Subsequently, the intracellular relay of the death signal activates effector caspase-3 to digest numerous proteins (Thorburn, 2004), leading to the apoptosis of target cells. When engaged with target cells, NK cells undergo rapid degranulation, secreting pore-forming perforins and pro-apoptotic effectors, such as granzymes (serine proteases), into immunological synapses (Voskoboinik et al., 2006), causing the death of proximal targets.

Aside from exerting their natural cytotoxicity, NK cells bridge innate and adaptive immunity. NK cells can produce proinflammatory cytokines (IFN-γ and TNF) and chemokines (CCL2, CCL3, CCL4, CCL5, CXCL8, and CXCL10) when stimulated with tumor cells expressing activating ligands (Fauriat et al., 2010), tilting their surroundings to a more hostile environment. In addition to the ‘missing-self hypothesis’ of NK cell activation, NK cells process antibody-dependent cellular cytotoxicity (ADCC). In other words, NK cells recognize antibody-opsonized target cells and become activated to exert their lytic mechanisms.

Immunotherapy emerges as a promising treatment for cancer patients. The essence of it is to reprogram and reactive the immune system in cancer patients to eliminate tumors, with approaches like blocking of the ‘brake’ on immune cell activation and administration of enhanced cell effectors. Immune effectors like cytotoxic T cells and NK cells are popular targets due to them being indigenously wired to tumor clearance. For example, anti-PD-1 therapy and anti-CTLA4 therapy blocks the deactivation interaction between cytotoxic T cells and tumor targets. However, some patients experienced relapses in response to anti-PD-1 antibody treatment via the loss of IFN-γ response and antigen presentation (Zaretsky et al., 2016). A genetic screening reveals that cancer cells with disrupted MHC-I or IFN-γ related genes are preferably killed by murine NK cells instead of cytotoxic T cells (Freeman et al., 2019). Administration of T cells modified with chimeric antigen receptors (CARs) promotes specific T-cell recognition of tumor antigens. CAR-T cells exhibit impressive clinical efficacy against refractory B cells but accompanied by safety concerns like cytokine release syndrome (CRS) and neurological events (Neelapu et al., 2017). Autologous CAR-T cells are safe with durable effects, but with technical difficulties and null ‘off-the-shelf’ potential (Locke et al., 2019). Adoptive transfer of NK cells between HLA-mismatched donors and recipients is demonstrated to be free of CRS (Bachanova et al., 2018; Miller et al., 2005; Ruggeri et al., 2002; Shaffer et al., 2016). Without need for prior sensitization, NK cells could be effective in targeting cancer cells that evaded T-cell immunity and possess higher ‘off-the-shelf’ capacity.

This Review will discuss the hurdles of NK cell immunotherapy, summarize the current trends in engineered NK cells for cancer therapy, and then discuss the prospects of integrating stem cell technology with engineering techniques.

## The status quo of adoptive NK cell therapy

Adoptive transfer of NK cells is a type of immunotherapy that follows this regimen: lymphodepletion by chemicals or radiation before the infusion of purified CD3−/CD56+ NK cells, followed by injection of immunostimulatory cytokines, commonly interleukin-2 (IL-2) and interleukin-15 (IL-15). The treatment is used against hematological malignancies (Bachanova et al., 2018) and solid tumors (Geller et al., 2011). Regardless of haploidentical or KIR-HLA mismatch, the treatment is safe with no signs of graft-versus-host disease (GvHD), CRS and neurotoxicity in recipients (Bachanova et al., 2018; Miller et al., 2005; Ruggeri et al., 2002; Shaffer et al., 2016) (Table 1).

**Table 1.**
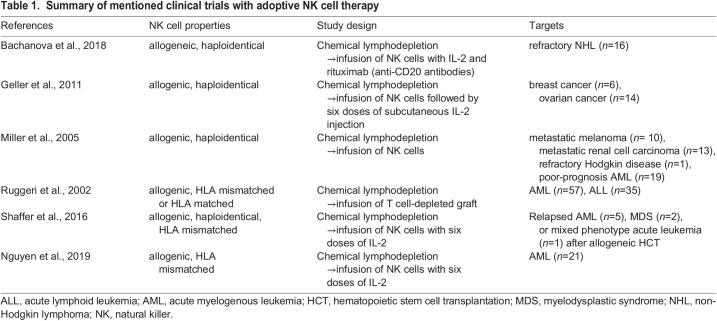
Summary of mentioned clinical trials with adoptive NK cell therapy

Poor *in vivo* persistence of infused NK cells is a bottleneck in adoptive NK cellular therapy (Nguyen et al., 2019; Shaffer et al., 2016). Following the mentioned regimen, allogenic haploidentical NK cells expand transiently post-infusion (between 7 and 14 days post-infusion), then gradually decline (Bachanova et al., 2018; Geller et al., 2011; Miller et al., 2005; Nguyen et al., 2019). Haploidentical NK cells level drops below 1% in peripheral blood (PB) 4 weeks after infusion in 61% of patients with acute myelogenous leukemia (AML) (Nguyen et al., 2019). The gradual drop in infused NK cells is hypothesized to be the action of regulatory T cells (Tregs) and myeloid-derived suppressor cells (MDSCs), hinted at by the negative correlation between NK cell proliferation and circulating Treg and MDSC levels (Bachanova et al., 2018), and the drop in circulating NK cell levels coinciding with the elevation in Tregs (Geller et al., 2011). The low *in vivo* persistence limits the clinical efficacy of the treatment and potentially accounts for the unaltered relapse incidence, event-free survival and overall survival, compared to those of the patient cohort receiving solely chemotherapy (Nguyen et al., 2019).

Administration of proinflammatory cytokines (either IL-2 or IL-15), which promotes the expansion of NK cells, is commonly employed to address the persistence issue, yet both are associated with toxicities. Infusing low doses of IL-2 in patients elevates the absolute number of CD56^bright^ NK cells by at least 7-fold (Fehniger et al., 2000). However, IL-2 promotes the *in vivo* expansion of CD4+CD25+Foxp3+ Tregs (Zorn et al., 2009), limiting the availability of circulating IL-2 for donor NK cells and potentially reducing NK cells persistence via Treg suppression. IL-15 expands and activates NK cells *in vivo* as well, proven by the 38-fold increase in circulating NK cells, and enhances the cytotoxic capacity of CD56^bright^ NK cells in patients receiving a continuous intravenous infusion of human recombinant IL-15 (Dubois et al., 2017). Of note, endogenous IL-15 production correlates with better clinical responses (Miller et al., 2005; Shaffer et al., 2016). Despite its effective impact on NK cells *in vivo* expansion, high doses of IL-2 (Klapper et al., 2008) or IL-15 (Miller et al., 2018) cause morbidity and severe adverse events (grade 3 or 4), and the circulating NK cell number drops upon withdrawal of cytokine treatment (Miller et al., 2018), hence rendering cytokine injection a non-sustainable option.

Oxidative stress is another challenge for immune cells' functionality in the tumor microenvironment (TME). By definition, oxidative stress refers to the imbalance between reactive oxygen species (ROS) production and anti-ROS mechanisms, causing a net increase in ROS (Aboelella et al., 2021). ROS are constantly generated as a by-product of increased metabolic activity of cancer cells and activated immune cells like granulocytes, macrophages and MDSCs. The direct impact of oxidative stress on immune cells varies among cell types and is dependent on the ROS level, which is discussed extensively elsewhere (Aboelella et al., 2021). Here, we focus on evidence arguing the effect of ROS on NK cells to justify the need for therapeutic intervention. NK cells have long been found to be susceptible to oxidative stress. Exposure of NK cells to hydrogen peroxide (H_2_O_2_), a type of ROS secreted by activated monocytes, downregulates surface CD16 expression and impairs the cytotoxicity of NK cells (Kono et al., 1996). Additionally, a high ROS level reduces NK cell infiltration into solid tumors. Infiltration of NK cells into tumor tissue of 29 non-small cell lung cancer (NSCLC) patients is significantly and negatively correlated with the frequency of NK cells with a high intracellular ROS level (Yang et al., 2020). Similarly, oxidative stress may be reversed by priming NK cells with IL-15. This can scavenge ROS via inducing the intracellular thioredoxin pathway and ectopic thiol expression, improving *in vitro* infiltration into the lung adenocarcinoma sphere (Yang et al., 2020). However, the resistance effect mediated by IL-15 also disappears once withdrawn.

Instead of administrating cytokines systematically, there are more attempts at modifying NK cells themselves to enhance NK cell activity in a more controlled and localized manner. An urge to modify NK cell *in vivo* persistence and effector function leads to numerous genetic engineering projects. Here, we group some recent projects into two directions: non-specific enhancement of NK cell activity and confer of specificity to NK cells.

## Non-specific functional enhancement

**Fig. 1.**
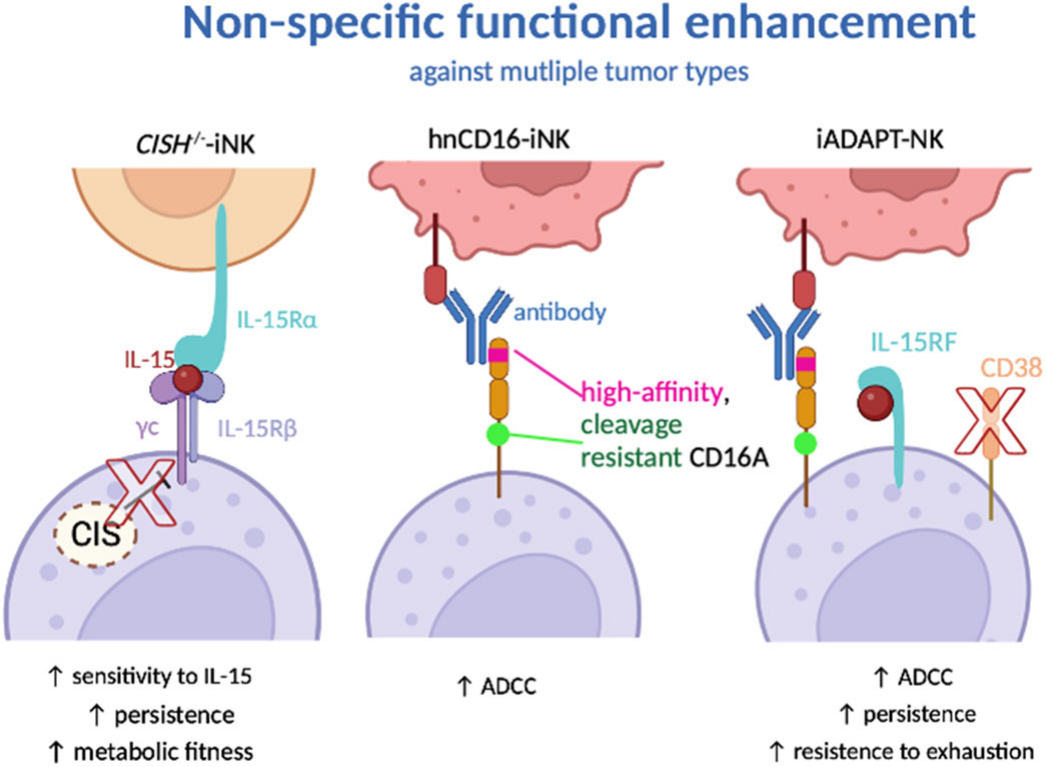
**Genetic modification to enhance natural killer (NK) cell functionality.** A schematic diagram summarizing the nomenclature, modification and corresponding functional consequences. ‘↑’ indicates the functional enhancement caused by the genetic modification. *CISH^−/−^* induced pluripotent stem cell-derived natural killer (iNK) cells knock out CIS, so its intrinsic inhibition of IL-15 signaling is removed. When IL-15 is *trans*-presented by neighboring cells and binds to NK cells' indigenous γc/IL-15Rβ receptors, *CISH^−/−^* iNK cells respond with higher sensitivity. hnCD16-iNK introduces an artificial high-affinity and cleavage-resistant CD16A to strengthen the antibody-dependent cellular cytotoxicity (ADCC) capacity of NK cells. iADAPT NK cells inherit the high-affinity, cleavage-resistant CD16A, and include two additional modifications, IL-15–IL-15Rα fusion protein (IL-15RF) and CD38 knockout.

We define non-specific functional enhancement as genetic engineering that boosts NK cell function non-specifically, so it is applicable in scenarios against multiple types of cancers (Fig. 1).

### Increasing sensitivity to cytokines

Instead of continuously administering IL-15, an approach is to knock out the negative regulators of proinflammatory cytokine signaling. *CISH* encodes cytokine-inducible SH2-containing protein (CIS), which is a cytokine-inducible negative regulator of JAK/STAT signaling (Delconte et al., 2016). The JAK/STAT signaling activated by IL-15 in NK cells can induce cytotoxicity (Gotthardt et al., 2014). CIS is rapidly induced in murine NK cells from IL-15 treatment, and the induced CIS, in turn, inhibits JAK1 by targeting it for proteasomal degradation, building a classic negative-feedback loop (Delconte et al., 2016). A recent approach depletes *CISH* in human induced pluripotent stem cells (hiPSCs) using CRISPR/Cas9 technology and subsequently differentiates the modified iPSCs into *CISH-*knockout (*CISH^−/−^*) iPSC-derived NK (iNK) cells, followed by expansion with artificial antigen-presenting (aAPC) feeder cells (Zhu et al., 2020a,b).

*CISH^−/−^* iNK cells expand and display *in vitro* short-term and long-term cytotoxicity, IFN-γ production and degranulation capacity against multiple tumor targets (K562, MOLM and SKOV3 cells) at IL-15- or IL-2-limiting conditions. MOLM-13 AML xenograft mouse models treated with *CISH*^−/−^ iNK cells (*n*=5) have better tumor control, of which 3/5 achieved complete tumor clearance, with a higher survival rate (vs 0% of iNK; *P*<0.001) 120 days post-infusion. More importantly, the human NK cell level in PB 2 weeks after *CISH*^−/−^ iNK treatment is higher than in the iNK group (38.2±4.3 vs 12.0±2.8 cells/µl; *P*<0.01). The knockout results in an elevated rate of glycolysis and oxidative phosphorylation compared to that in iNK and PB-NK cells, providing the basis for meeting the energy requirements of executing effector functions.

This approach successfully enhances NK cells’ *in vitro* and *in vivo* anti-tumor activity, persistence and metabolic fitness by increasing the sensitivity to IL-15. Therefore, low-dose treatment of IL-15 can augment NK cell function comparably while avoiding the side effects associated with high-dose treatment (Miller et al., 2018). Despite the *in vivo* efficacy and persistence of *CISH^−/−^* iNK cells not being tested in an IL-2-limiting model, perturbation of a single gene provides a promising solution to both persistence and effector limitations.

### Protecting NK cells from immune checkpoint regulation

CD16 (FCγRIII) is an extracellular receptor that binds to the Fc portion of IgG1 and IgG3 antibodies (Nimmerjahn and Ravetch, 2008), and the isoform, CD16A, is expressed in mature NK cells to induce ADCC activity. It is a potent activating receptor of NK cells, yet stimulating it with antibodies again reduces the amount of released perforin from stimulated NK cells (Srpan et al., 2018). CD16A surface expression is downmodulated when NK cells are activated *in vitro* by various means (Lajoie et al., 2014) and observed on NK cells isolated from patients with ovarian carcinoma as well (Lai et al., 1996). This phenomenon is caused by the shedding of CD16A ectodomain, mediated by a disintegrin and metalloprotease-17 (ADAM17) (Romee et al., 2013) and membrane-type 6 matrix metalloproteinase (MTP-MMP) (Peruzzi et al., 2013). ADAM17 is expressed constitutively on the NK cell membrane (Lajoie et al., 2014), rapidly cleaving CD16A in a *cis* manner. Recovery of CD16 requires 1 or 2 weeks (Goodier et al., 2016). Shedding of CD16A on NK cells limits its ADCC capacity, therefore, representing an immune checkpoint to limit the anti-tumor efficiency.

Engineering hiPSC-derived NK cells to constitutively express a high-affinity and non-cleavable form of CD16A (Zhu et al., 2020a,b), with a similar NK cell generation protocol described earlier, solves the challenge. The modified form of CD16A consists of two mutations: a 158V mutation inspired by a natural allelic variant (Koene et al., 1997) that accounts for higher affinity to IgG; and an S197P mutation accounting for its cleavage resistance against ADAM17 (Jing et al., 2015). These modified NK cells are termed hnCD16-iNK.

hnCD16-iNK cells alone exhibit minimal NK cell activation and cytotoxicity, but the combination treatment of hnCD16-iNK cells and therapeutic monoclonal antibody (mab) shows higher cytotoxicity against multiple tumors (Raji, SKOV-3, Cal 27) (Zhu et al., 2020b). The *in vivo* ADCC of hnCD16-iNK is proven in xenograft models against human B-cell lymphoma, systemic lymphoma (Raji cells) and even solid ovarian carcinoma (SKOV3 cells) (Zhu et al., 2020b). Infusing mab with hnCD16-iNK cells declines tumor burden to a larger extent than sole antibody or hnCD16-iNK treatment, and mediates the long-term survival of a SKOV3 xenograft model (150 days post-transplant; vs anti-HER2, *P*=0.004) (Zhu et al., 2020b). However, the circulating NK cell population with single-dose treatment reduces to baseline 20 days post-infusion. Tumor regression caused by the combination treatment does not persist after 10 days, and the tumor burden at day 25 is indeed comparable with that of other groups (co-administration of iNK/PB-NK with anti-CD20 mab) (Zhu et al., 2020b).

Interestingly, this ‘proof-of-principle’ test of combining engineered NK cells and therapeutic monoclonal antibodies demonstrates a more holistic reprogramming approach. hnCD16-iNK has superior *in vitro* and *in vivo* ADCC than PB-NK or unmodified iNK (Zhu et al., 2020b). Flexibility to target multiple tumors can be achieved via administration of corresponding therapeutic antibodies, hence potentially tackling the more immunosuppressive solid tumors, as suggested by *in vitro* and *in vivo* superior ADCC against SKOV-3, ovarian adenocarcinoma (Zhu et al., 2020b). Meanwhile, crude selectivity can be achieved with the corresponding therapeutic mab (Zhu et al., 2020b). The shortcoming is that a single dose of infusion provides transient control. Genetic engineering overcomes an immune checkpoint of NK cells and equips them with more effective anti-tumor capacity, but this is not enough.

### Triple-gene modifications mimic adaptive NK cells

Continuing the prior genetic engineering project, three genetic modifications on iNK cells produce a version of enhanced NK cells named iADAPT NK cells (Woan et al., 2021), which harness several attributes resembling adaptive NK cells, including a long half-life and being elite in ADCC and IFN-γ production (Stary and Stary, 2020). These genetic modifications equip iNK cells with a high-affinity, non-cleavable CD16A as a continuation of the previous attempt, a membrane-bound IL-15 fused with IL-15 receptor α subunit (IL-15RF), and CD38 knockout. The membrane-bound IL-15RF creates a *cis* version of *trans*-presentation of IL-15, a phenomenon describing the stimulation of IL-15 signaling from a complex constitutive of IL-15 and IL-15 high-affinity binding proteins (i.e. IL-15Rα) formed intracellularly (Anderson et al., 1995; Stonier and Schluns, 2010). IL-15 and IL-2 bind to a shared β/γC subunit in their receptor complex, but it can induce a distinct response due to the *trans*-presentation effect (Waldmann, 2006). *Trans*-presentation is more prevalent as IL-15 is rarely produced by NK cells themselves, despite NK cells expressing IL-15Rα transcripts (Rosmaraki et al., 2001). The effect of this complex binding to the β/γC receptor complex expressed on NK cells is more potent than that of soluble IL-15 (Huntington et al., 2009). Knocking in the construct allows NK cells to *cis* present IL-15Rα/IL-15 to the β/γC receptor complex. Hence, when compared to the systemic administration of IL-15, it enhances NK cells' persistence with more localized and potent regulation of IL-15 signaling.

CD38 knockout is a novel take on modifying stem cell-derived NK cells to be more similar to adaptive NK cells. CD38 is an ectoenzyme that exerts both NADase and ADP-ribosyl cyclase activity, which is an important regulator of NAD+ levels to control various metabolic activities (Malavasi et al., 2008). Recently, CD38 was found to be enriched in a dysfunctional CD8+ T-cell subset, which failed to generate memory or display effector functions like IFN-γ production in two independent scenarios (Katsuyama et al., 2020; Verma et al., 2019), while CD38 knockdown enables dysfunctional T cells to regain their effector capability (Verma et al., 2019). As adaptive NK cells share some common epigenetic and transcriptional features with CD8+ T cells (Lau et al., 2018), the group hypothesized that CD38 knockout augments NK cell resistance to suppressive TME.

iADAPT NK cells killed MM.1R myeloma cells in three rounds of 48-h coculture, even without aid from the anti-CD38 antibody, daratumumab (Woan et al., 2021). Co-administration of iADAPT NK cells and daratumumab achieved better tumor control in HL-60 AML cell-xenografted mice (*n*=5) than sole antibody or iADAPT NK cell treatment, or even in more aggressive MM.1S-xenografted mouse models (*n*=5). Without exogenous cytokines, NK cells are detectable in PB of an HL-60 AML xenograft model (*n*=5) 40 days post-injection, doubling the *in vivo* persistence of hnCD16-iNK (21 days post-treatment) (Woan et al., 2021).

With triple-gene modifications, this approach generates iADAPT NK cells with superior *in vivo* persistence, *in vitro* and *in vivo* ADCC, and *in vitro* resistance to exhaustion in the absence of exogenous cytokines. This is particularly compatible with the combination treatment of daratumumab against leukemia, as the daratumumab can be selective to CD38 expressed on myeloma cells. Although the killing activity is primarily accessed in leukemia, the retained innateness and enhanced persistence can theoretically effectively eliminate tumors of choice in cytokine-limiting conditions. The cytokine autonomy conferred by IL-15RF is genuinely a breakthrough to overcoming the hurdle of hnCD16 iNK, evading the adverse events associated with IL-2 (Klapper et al., 2008) or IL-15 (Miller et al., 2018) dosing.

Altogether, the three applications we dichromatically classified as non-specific functional enhancements – *CISH*^−/−^ iNK cells, hnCD16-iNK cells and iADAPT NK cells – prove how genetic modifications equip NK cells with versatile anti-tumor capability against multiple tumors. These genetic modifications either enhance persistence, enhance effector functions or achieve both through different paths. More considerations have been taken in complying adoptive transfer of modified NK cells with existing treatment, i.e. with therapeutic antibodies, to represent a more holistic immunotherapeutic approach against cancers.

## Conferring specificity

**Fig. 2.**
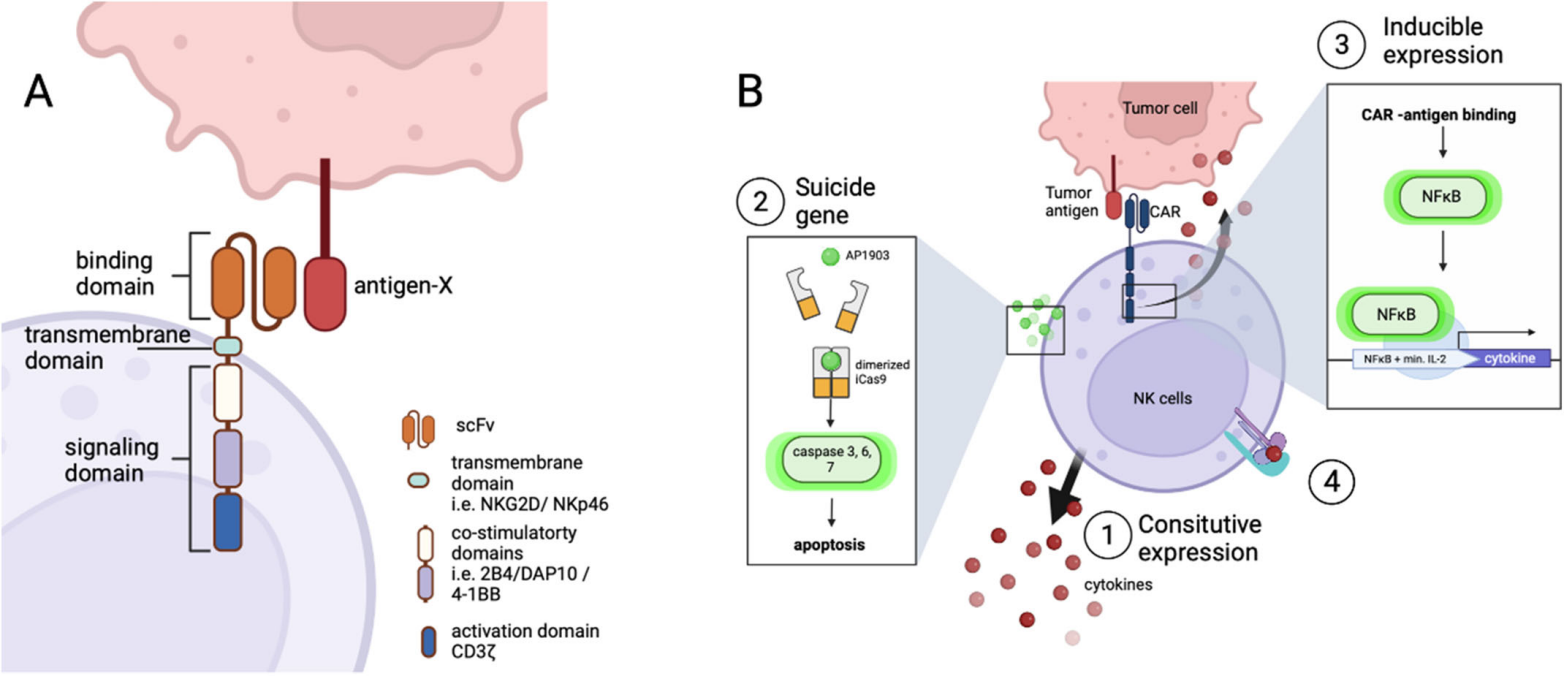
**Principle and applications of CAR-NK cells.** (A) General design of the chimeric antigen receptor (CAR). The current CAR construct can be generalized into three domains: the extracellular binding domain, a transmembrane domain and an intracellular signaling domain. The binding domain comprises a single-chain variable fragment (scFv) to bind to a predefined antigen. The transmembrane domain is usually adapted from the transmembrane domain of activating receptors on NK cells. The signaling domain consists of co-stimulatory domain, adapted from co-stimulatory receptors as well, and a CD3ζ activation domain. (B) Strategies to regulate transgene expression. Additional payloads are introduced into CAR-NK cells to improve clinical outcomes. Transgenic expression of cytokines is used as an example to illustrate some regulatory strategies: (1) cytokine transgene is downstream of a constitutive promoter with uncontrollable secretion; (2) addition of a drug-inducible suicide gene into CAR-NK cells eliminates infused cells at any time point; (3) inducible expression of transgene allows cytokine secretion when CAR-NK cells are activated by binding to targets; (4) creation of a membrane-bound form of cytokines.

Another major area of interest to genetically modified NK cells is to confer specificity towards a single target, mainly through the addition of a CAR construct (Fig. 2). Despite the proven safety to transfer HLA-mismatched NK cells (Bachanova et al., 2018; Miller et al., 2005; Ruggeri et al., 2002; Shaffer et al., 2016), theoretically, off-target killing is possible due to their innate cytotoxicity. In addition, NK cell effector functions are hindered in the immunosuppressive TME. To kill two birds with one stone, the endowment of a CAR confers specificity and activates NK cells continuously in the presence of an assigned target.

The general design of a CAR consists of three modules: an extracellular antigen-recognition receptor, a transmembrane linker and an intracellular activation domain. The extracellular construct usually comprises a single-chain variable fragment (scFv) that binds to a specific epitope, recognizing a predefined target. The transmembrane domain connects the two modules spatially. The intracellular part of this artificial construct includes signaling domains and co-stimulatory domains to initiate activation pathways.

The generation of an NK cell-specific CAR construct is described in the trial of mesothelin-specific CAR-NK cells from iPSCs (Li et al., 2018). The extracellular domain part remains to be an scFv, but the transmembrane domain and co-stimulatory domains are adapted from NK cell-activating receptors, including NKG2D, CD16, DAP10 (co-stimulatory domain of NKG2D), 4-1BB and 2B4 [both are co-stimulatory receptors with intra-cytoplasmic immunoreceptor tyrosine-based activation motifs (ITAMs)] (Yoon et al., 2015), and a CD3ζ domain shared by T cells' CD3 and NK cells' CD16.

### Improving CAR-NK with cytokine transgenes

To overcome the persistence limitation of adoptively transferred NK cells, one approach is to combine a CAR with the IL-15 transgene. As mentioned before, injection of IL-15 promotes *in vivo* expansion of NK cells, but it poses an immense financial burden to patients. If we require the cytokine anyway, why not just fuse the transgene with the CAR construct, allowing NK cells to persist without exogenous cytokines?

The fusing of the IL-15 transgene downstream of the CAR construct is proven to be effective against various types of tumors. Recent work on fusing CD123-specific CAR-NK with the IL-15 transgene against hematological malignancies activates NK cells, yet is associated with systemic toxicities (Christodoulou et al., 2021). Human primary NK cells from three independent donors are expanded with feeder cells, then transduced with a retroviral vector of CD123-CAR/2B4/CD3ζ and a downstream internal ribosome entry site (IRES) fused with human IL-15 transgene, allowing co-expression of IL-15 and CAR construct under the constitutive promoter.

The 2B4/CD3ζ/soluble interleukin-15 (sIL-15) CAR-NK cells show comparable tumor control against CD123+ MV-4-11 cells *in vivo* to 2B4/CD3ζ CAR-NK while displaying an exponential increase in the circulating cell count (35 days; *n*=5-7). However, the expansion advantage of 2B4/CD3ζ/sIL-15 CAR-NK cells does not translate into enhanced survival. The cohorts receiving either sIL-15-NK cells (complete death around day 23 post-infusion) or 2B4/CD3ζ/sIL-15 CAR-NK cells (complete death around day 35 post-infusion) have a shorter survival time. Although constitutive IL-15 expression in CAR-NK can enhance effector phenotypes, as would have been expected from clinical benefits observed from the injection of IL-15 (Dubois et al., 2017), the lethality observed in mice urges for more stringent control of transgene expression.

In this regard, here we present some examples of how to control cytokine expression temporally in CAR-NK cells. Liu et al. (2018) reported the transduction of an inducible caspase 9-based (iC9) suicide gene into CD19-CAR/IL-15-expressing NK cells isolated from cord blood (iC9/CAR.19/IL-15 CB-NK). IL-15 production increaseds significantly when challenged with CD19+ chronic lymphocytic leukemia (CLL) targets with minimal secretion in the absence of CD19+ targets. Administration of dimerizer (AP1903) *in vivo* successfully triggers the suicide response to reduce CAR-expressing NK cells in PB (98%→11%) and tissues within 3 days. This drug-inducible regulation avoids the adverse outcomes associated with cytokine expression, but the premature termination of transferred NK cell activity limits the persistence advantages.

A more optimal control strategy is the use of an inducible promoter to regulate cytokine expression. A recent ‘proof-of-principle’ work focused on transducing NK-92 and primary NK cells with a vector containing constitutively expressed disialoganglioside GD2-specific CAR and inducible human IL-12 (hIL-12) regulated by NFκB (iIL-12/GD2.CAR-NK cells) (Rudek et al., 2021). NFκB is a signaling molecule expressed in activated NK cells (Goodier et al., 2016). With this design, CAR-mediated antigen recognition and activation trigger the endogenous activation signaling cascades, subsequently upregulating NFκB, leading to hIL-12 transgene expression. In other words, hIL-12 is only produced when CAR-specific antigens are presented. Coherent with the theory, no hIL-12 is produced when iIL-12/GD2.CAR-NK cells are at rest or co-cultured with GD2-lacking target (HT1080), but significantly secreted when co-cultured with GD2+ targets, GD2+ HT1080, SH-SY5Y and patient-derived glioblastoma. Upon target stimulation, IFN-γ and IL-2 production are significantly enhanced in iIL-12/GD2.CAR-NK cells, which is in line with the function of IL-12 (Zhang et al., 2008). The iIL-12/GD2.CAR-NK cells are not tested *in vivo*, so we cannot conclude whether this design improves the *in vivo* persistence of CAR-NK cells. Yet, with the modular nature of this design, we can foresee the replacement of hIL-12 transgenes with other cytokines like IL-15 to produce CAR-NK cells with inducible cytokine production.

Spatial regulation may be achieved via membrane-bound cytokine expression. NK cells express the high-affinity IL-15-binding protein IL-15Rα (Rosmaraki et al., 2001). Theoretically, the engineered membrane-bound IL-15 can *trans-* or *cis*-present to itself or surrounding CAR-NK cells, achieving a localized enhancement of persistence of infused products. CD19-CAR-NK cells with ectopic IL-15 expression and iC9 suicide gene have gained clinical success against CD19+ lymphoma, with 7/11 recipients demonstrating complete remission, and proven safety, whereby none of the recipients developed symptoms of CRS, GvHD and neurotoxicity. CAR-NK cells are also detectable 12 months after infusion (Liu et al., 2020). Another ongoing phase 1 clinical trial using NKX019, a CD19-targeting CAR-NK with membrane-bound IL-15 (NCT05020678), which is expected to end in July 2023, further informs about the persistence and safety of this approach.

The fusing of a cytokine transgene downstream of the CAR construct represents a feasible strategy to overcome the bottleneck observed in previous CAR-NK trials. Constitutive expression of cytokines causes lethality, despite its evident benefits. Thus, extra control mechanisms are anticipated, such as the incorporation of a suicide gene, utilization of an inducible promoter, or fusion with a membrane-bound form to spatially and temporally regulate the balance between anti-tumor effects and safety.

### CAR-NK cells with resistance against oxidative stress

Another bottleneck that hinders the *in vivo* anti-tumor efficacy of infused NK products is the high oxidative stress in the TME. To combat the oxidative stress in the breast cancer microenvironment, primary NK cells and NK92 cells are genetically modified into PD-L1-CAR-NK cells overexpressing *PRDX1* via a lentiviral transduction system (Klopotowska et al., 2021). As mentioned before, priming NK cells with IL-15 mediates transient resistance to oxidative stress in the TME (Yang et al., 2020). *PRDX1* encodes an antioxidant peroxidase that reduces H_2_O_2_ to water and alcohol and is significantly upregulated in IL-15-primed NK cells transcriptionally and translationally (Klopotowska et al., 2021). Under H_2_O_2_ -mediated oxidative stress, the PD-L1-CAR-NK cells demonstrate impaired killing activity against triple-negative breast cancer MDA-MB-231 cells, which was rescued with *PRDX1* overexpression in PD-L1-CAR-NK cells, equipping CAR-NK cells with resistance against H_2_O_2_ -mediated oxidative stress in IL-15-independent settings.

Recent genetic engineering projects enhance NK cells' functionality by enhancing them non-specifically, with or without conferring capability to target a specific antigen. Non-specific effector enhancement further manifests the ‘off-the-shelf’ capacity. Following this direction, not only NK cell immunotherapy is applicable in HLA-mismatched donors and recipients, but also applicable in multiple types of tumors. In some trials, specific activation of NK cells can be conferred with exogenous aid, i.e. monoclonal antibodies, but itself will not show specificity. Therefore, off-target killing may be a concern in clinical settings.

Regarding conferring specificity to NK cells by the endowment of CAR construct, precision is emphasized, so NK cells are often only effective against tumors bearing predefined antigens. The modular nature of CAR does permit flexibility to target multiple tumors, but it remains technically troublesome to tailor-make CAR-NK cells and identify antigens with the best clinical outcomes. No matter which approaches, the major goal of genetic engineering is resolving bottlenecks encountered in clinical trials of adoptive NK cell therapy. One prominent direction is to exploit the benefits of IL-15 administration to resolve the persistence and oxidative stress limitations while stressing cytokine independence. Thus, clinical application of these genetically modified NK cells can evade the adverse events associated with high-dose IL-15 administration and improve recipients' burden on continuous cytokine injections.

### The prospect of integrating stem cell technology

**Fig. 3.**
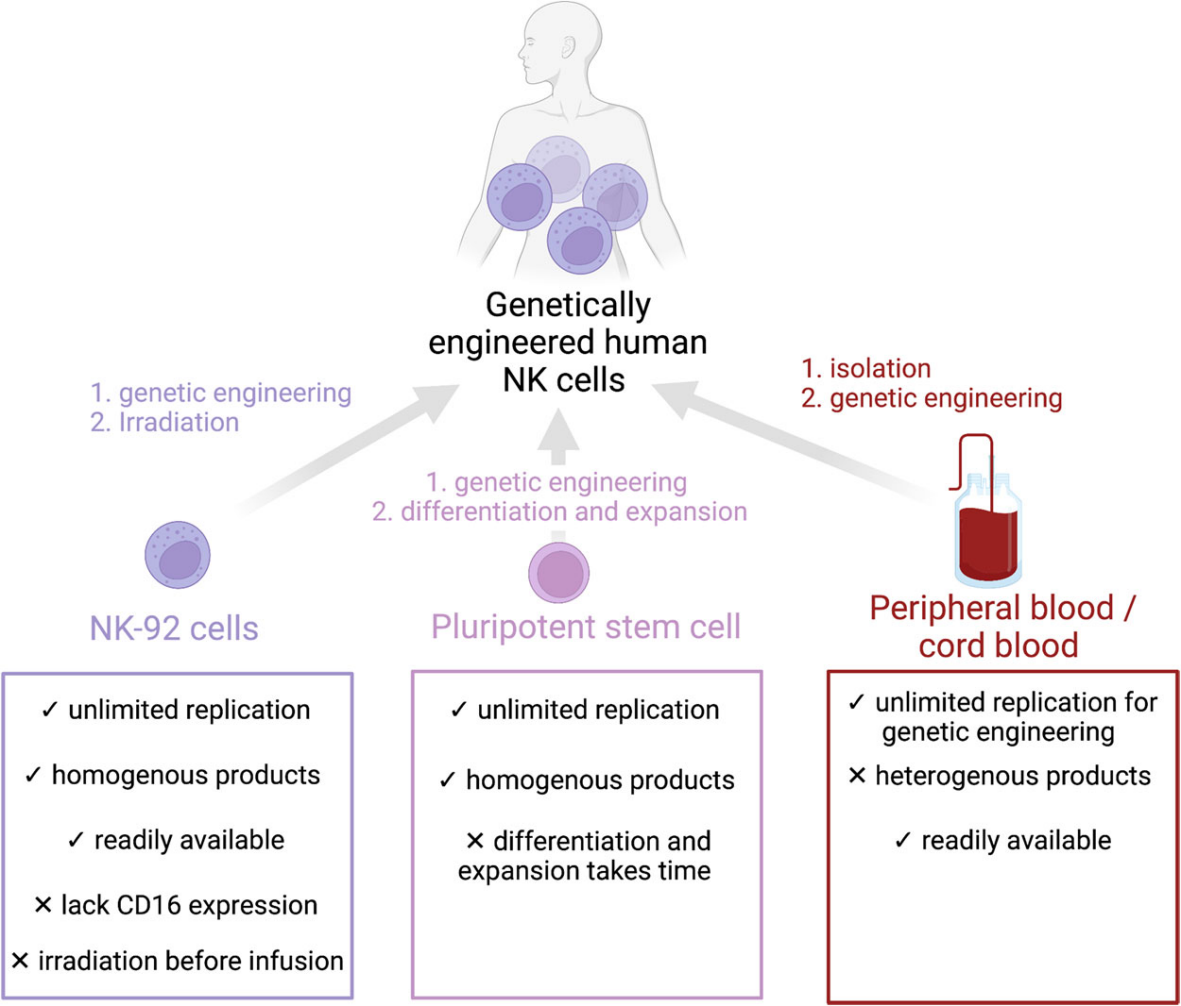
**Comparison between common sources of NK cells.** ‘√’ represents advantage for genetic engineering; ‘×’ represents limitation. The steps to prepare the NK cells for genetic engineering and infusion are listed.

The ideal source of NK cells for genetic engineering projects is vital to optimizing clinical practices, yet each source has its pros and cons (Fig. 3). Human NK cells isolated from donated PB or cord blood, commercial human NK cell lines (NK-92 cells or their derivatives) and pluripotent stem-cell-derived NK cells are satisfactorily effective allogenic sources. Here, we compare and contrast the three sources to discuss how stem cell-derived NK cells outcompete others to produce clinically graded genetically engineered NK cells.

Human NK cells can be isolated from donated PB and cord blood with a rapid large-scale automated method, mostly by two-step magnetic-activated cell sorting (MACS) of CD3-CD56+ cells from the mononuclear cell product by leukapheresis (Iyengar et al., 2003). The obtained NK cells can be further expanded with feeder cells (Christodoulou et al., 2021). The infusion of human primary NK cells is safe and a very popular source for the adoptive transfer of NK cells in clinical trials (Bachanova et al., 2018; Miller et al., 2005; Ruggeri et al., 2002; Shaffer et al., 2016). Yet, the use of PB competes with other medical usages of blood.

NK92 cells are commercially available permanent human NK cell lines isolated from large granular lymphocytes (LGLs) with the phenotype of CD56^bright^, CD45+ and CD3− (Gong et al., 1994). Culturing of this cell line is dependent on IL-2, so there are some engineered IL-2-dependent NK92 cell lines, such as NK92mi cell lines (ATCC). It is very effective against K562 cells and Daudi cells (Gong et al., 1994) and was proven to be free from grade III or IV toxicities in clinical trials (Tang et al., 2018; Williams et al., 2017). The winning edge of this source is how readily available it is. NK92 cells are commercially available ‘off-the-shelf’ products to meet multi-dosing needs. Their unlimited replicative potential renders them more compatible with the retroviral transduction system (Xu et al., 2019). However, the NK-92 cells are irradiated before infusion to limit their replicative potential, so they can only persist at most for 1 week *in vivo* (Tang et al., 2018).

Pluripotent stem cells are characterized by the ability to self-renew and differentiate to all types of cells, apart from cells in extra-embryonic lineages. To use stem cell-derived genetically engineered NK cells, pluripotent stem cells are engineered via the method of choice, including, but not limited to, lentivirus transduction and CRISPR/Cas9 technology, to incorporate the transgene and selectable markers. Again, their unlimited replicative potential permits the use of the retroviral transduction system. The stem cells bearing the transgene are selected out with the addition of selective agents in medium or sequencing. Then, the stem cells dissociated to a single-cell level will differentiate into NK cells, following an established protocol (Zhu and Kaufman, 2019), and expand with feeder cells (Denman et al., 2012; Zhu et al., 2020a,b).

The ability to produce highly homogenous NK cell products from engineered pluripotent stem cells is a major advantage over terminally differentiated sources of NK cells. The resulting products are highly homogeneous in terms of surface receptor expression (Zhu and Kaufman, 2019) and the introduced genetic modifications, as they are derived from the same stem cell clone. Meanwhile, blood-derived NK cells have their intra- and inter-individual diverse arrays of germline-encoded activating and inhibitory receptors. Although genetically modified cells can be selected, the variance among different batches will be considerably large (Christodoulou et al., 2021), rendering repeat dosing unreproducible. NK92 cells are deficient in some key activating receptors, so they are less effective than stem cell-derived NK cells. For instance, NK92 cells lack CD16 expression (Gong et al., 1994; Srpan et al., 2018), which affects the ADCC mechanism of NK cells. On the other hand, stem cell-derived NK cells express many activating receptors, such as NKG2D, NKp46, NKp44, FasL and CD16 (Zhu et al., 2020a,b). The homogeneous presence of NK cell receptors allows stem cell-derived NK cells to be ideal for clinical usage.

A limitation of stem cell-derived NK cells is their time-consuming production, which is detrimental in patients in need of an immediate cure. Following the production protocol, making NK cells from pluripotent stem cells takes 4 weeks, with an additional 2 weeks for expansion (Zhu and Kaufman, 2019). The dosage of cells/kg body weight recorded in some human trials of adoptive transfer of non-genetically manipulated primary NK cells ranges from 0.5×10^6^ to 2×10^7^, which can be collected within 1 day (Bachanova et al., 2018; Geller et al., 2011; Miller et al., 2005; Nguyen et al., 2019; Ruggeri et al., 2002; Shaffer et al., 2016). Following this differentiation protocol (Zhu and Kaufman, 2019), they can generate 2-20×10^6^ NK cells from one six-well plate. The *ex vivo* expansion by stimulating differentiated NK cells with membrane-bound IL-21-experessing irradiated K562 cells under the continuous supply of IL-2 sustains NK cell viability without telomere shortening for over 3 months (Denman et al., 2012; Zhu and Kaufman, 2019). Alternatives to feeder cells are irradiated K562 cells that express membrane-bound IL-21 and 4-1BBL (Zhu et al., 2020a,b), or membrane-bound IL-15 and 4-1BBL (Lapteva et al., 2012), and the latter have been proven to produce primary NK cells from PB mononuclear cells with good manufacturing practice (Lapteva et al., 2012). Additionally, the stem cell-derived NK cells retain their anti-tumor capability after cryopreservation (Woan et al., 2021; Zhu et al., 2020a,b). The expansion is thus highly desired for reducing the cost and enabling multi-dosing strategy, although fails to meet urgent needs like using primary NK cells.

NK cells collected from blood, pluripotent stem cell differentiation or NK92 cells undergo different degrees of genetic manipulation to enhance tumor response. The ideal gene delivery method has to be safe and efficient. Retroviral transduction is often used to introduce CAR constructs into expanding primary NK cells (Christodoulou et al., 2021; Liu et al., 2018; Rudek et al., 2021) and NK-92 cells (Klopotowska et al., 2021; Rudek et al., 2021). Being capable to infect both non-dividing and dividing cells (Naldini et al., 1996), lentivirus can also be employed to deliver PD-L1 CAR constructs into NK92 cells (Klopotowska et al., 2021), and high-affinity, cleavage-resistant CD16 sequences into iPSC-derived NK cells and primary NK cells (Zhu et al., 2020a,b). The feature of lentiviral and retroviral transduction is that they can integrate transgenes into the genome, achieving stable expression of exogenous constructs. However, the efficiency to transduce primary NK cells is generally low with retroviruses (Xie et al., 2020), i.e. the transduction efficiency with alpharetroviral vector into primary NK cells is around 30% (Rudek et al., 2021), and the random insertion of transgene potentiates harmful mutations (Xie et al., 2020). Therefore, non-viral alternatives are appealing to deliver the foreign construct. Electroporation is preferable to transduction due to its high efficiency and compatibility with most cell types (Potter, 2003). Despite the expression being transient, stable expression of the exogenous construct is viable by integrating the sequence into a specific location of the genome via homologous repair. For example, the CRISPR/Cas9 construct is delivered into iPSC-derived cells by electroporation-based nucleofection to perform a triple-genetic modification (Woan et al., 2021). The sequence of the donor plasmid for membrane-bound IL-15RF and hnCD16 was inserted into the genome with CRISPR/Cas9-mediated target insertion.

## Conclusion

Adoptive transfer of NK cells is a safe immunotherapeutic treatment against cancer, yet its effectiveness is limited by poor *in vivo* persistence and oxidative stress in the TME. Genetic engineering of NK cells enhances their persistence and lytic activity. We identified two major areas for genetic engineering, the non-specific enhancement of NK cell functionality and the endowment of specificity to NK cells. The prior is applicable against multiple types of tumors, while the latter allows NK cells to be extremely ‘on-target’. Both areas aim to exploit the benefits observed from the systemic administration of IL-15, making cytokine-autonomous cellular products to evade the adverse events associated with high-dose IL-15 treatment. The source of NK cells is diverse, but stem cell-derived NK cells are highly compatible with genetic engineering projects, due to their high homogeneity. Many of the reviewed trials are ‘proof-of-principle’ tests that demonstrate effective anti-tumor activity in mouse models. Future phase I clinical trials can investigate the safety of the genetically modified NK cells.
